# Distributed Reconstruction via Alternating Direction Method

**DOI:** 10.1155/2013/418747

**Published:** 2013-08-20

**Authors:** Linyuan Wang, Ailong Cai, Hanming Zhang, Bin Yan, Lei Li, Guoen Hu

**Affiliations:** National Digital Switching System Engineering & Technological R&D Center, Zhengzhou 450002, China

## Abstract

With the development of compressive sensing theory, image reconstruction from few-view projections has received considerable research attentions in the field of computed tomography (CT). Total-variation- (TV-) based CT image reconstruction has been shown to be experimentally capable of producing accurate reconstructions from sparse-view data. In this study, a distributed reconstruction algorithm based on TV minimization has been developed. This algorithm is very simple as it uses the alternating direction method. The proposed method can accelerate the alternating direction total variation minimization (ADTVM) algorithm without losing accuracy.

## 1. Introduction

Image reconstruction algorithms implemented in existing computed tomography (CT) scanners require projection data to be available in proportional space [1, 2]. However, in CT imaging of biological specimens, data collection at a large number of projection views may result in radiation-induced object deformation. Recently, methods based on the corresponding constrained total variation (TV) or *l*_1_-norm minimization have been widely studied in reconstruction from sparse-view data [3–9]. Sidky et al. [5] proposed the adaptive steepest descent projection onto convex sets (ASD-POCS) algorithm for CT image reconstruction. This method can realize exact-image reconstruction using fewer measurements. The alternating direction method (ADM) is an efficient approach for optimization problems. And the split Bregman-TV method based on alternating Bregman iterative approach was proposed and converged well in [8] as a solution for sparse-view CT reconstruction. A TV minimization iterative algorithm using the ADM based on augmented Lagrangian function was also proposed [10, 11]. Li et al. proposed a more robust and efficient algorithm nonmonotone alternating direction algorithm (NADA) [12] in 2012, which integrates alternating direction and nonmonotone line search. An alternating direction total variation minimization (ADTVM) algorithm for few-views reconstruction [13] was developed inspired by the literature [10–12]. The augmented Lagrangian function-based ADM is actually equivalent to the Bregman iterative method when the constraints are linear [14]. However, the expression in [13] is simpler than that in [8].

The CT image reconstruction problem is a large-scale problem. The ADTVM algorithm [13] is not directly suitable for distributed implementation. Boyd et al. [15] argued that the alternating direction method of multipliers is well suited for distributed convex optimization, in particular, for large-scale problems arising in statistics, machine learning, and other related areas. In this study, a distributed algorithm called distributed alternating direction total variation minimization (Dis-ADTVM) is developed using ADM. The proposed algorithm is as simple as the ADTVM algorithm and can accelerate the latter without accuracy loss.

## 2. Method

The imaging model can be approximated using the following discrete linear system:

(1) $$\begin{array}{c} p=W\vec{f}, \end{array}$$

where the vector *p* has length *N*_*d*_, which is the number of measured projection rays; the vector $\vec{f}$ has length *N*_*im*_, which is the number of expansion elements used in representing the object function$f(\vec{r})$; the system matrix *W* is a pixel-driven projection operator.

Sparse-views projection data are not sufficient for exact reconstruction. The problem we consider in this study is ill-posed. To solve linear system (1), we use a regularization method with anisotropic TV minimization, as follows:

(2) min⁡ ||f→||TV s.t.  p=Wf→,

where ${||\vec{f}||}_{TV}≜\sum_{j}{||D_{j}\vec{f}||}_{1}$ and *D*_*j*_ denotes the differential operator along direction *j*. In particular, *D*_1_ and *D*_2_ denote the horizontal and vertical differential operators, respectively, for two-dimensional form. *p* and *W* are separated in *i* along vertical direction as

(3) min⁡ ||f→||TV s.t.  pi=Wif→, i=1,2,…,N.

We consider a variant of (3) as follows:

(4) min⁡12∑i||Wif→i−pi||2+λ∑j||zj||1 s.t.  Djf→ i=zj,

where ${\vec{f}}_{i}$ denotes $\vec{f}$ in node *i* and ${\vec{f}}_{i}=\vec{f}$, *i* = 1,2,…, *N*, in node *i*. Its corresponding augmented Lagrangian function is

(5) LA(z1,z2,f→ i)=∑i(12||Wif→i−pi||2   +∑j(λ||zj||1+uijT(Djf→i−zj)      +ρ2||Djf→i−zj||2)),

where *u*_*ij*_ is Lagrange multiplier and the parameters *λ* and *ρ* are both used to balance the terms. The ADM is used to solve the problem that minimizes the augmented Lagrangian function; that is,

(6) f→ ik+1=argminf→i(12||Wif→i−pi||2+ρ2∑j||Djf→i−zjk+ujik/ρ||2),zjk+1=argminz(λ||zj||1+Nρ2||Djf→ k+1¯−zj+ujk¯/ρ||2),ujik+1=ujik+ρ(Djf→ ik+1−zjk+1).

The final algorithm of Dis-ADTVM can be expressed as

(7) f→ ik+1=(ρ∑jDjTDj+WiTWi)+ ×(WiTpi+ρ∑jDjT(zjk−ujik/ρ)),zk+1=max⁡{|Djf→¯k+1+ujk¯Nρ|−λNρ,0}×sgn⁡(Djf→¯k+1+ujk¯Nρ),ujik+1=ujik+ρ(Djf→ ik+1−zjk+1),

where $\vec{f}{\;}_{i}^{k+1}$ and *u*_*ji*_^*k*+1^ can be computed in node *i* and *M* ^+^ stands for the Moore-Penrose pseudoinverse of matrix *M*. Computing the pseudoinverse at each iteration is too costly to implement numerically, while we use NADA [12] to solve “*f*-subproblem” in (7).

The convergence analysis of ADTVM algorithm has been well analyzed in the literature [12], and the convergence proof of the distributed algorithms based on ADM can be found in [15, 16]. The iterative algorithms using the ADM based on augmented Lagrangian function decompose the optimization problem into some simple subproblems with closed form solution. Therefore, the algorithms are efficient and practical for the low cost in each iteration. The NADA algorithm enables taking full advantages of the low-cost minimization in “easy” direction and allows relatively quick and large steps in the “hard” direction. The distributed algorithms can distribute some computation to individual nodes; thereby, the algorithms reduce the running time through data distribution and computation. The proposed Dis-ADTVM algorithm in this paper integrates above advantages and its derivation and implementation are as simple as the ADTVM algorithm.

## 3. Numerical Results

### 3.1. Simulation Studies

We perform numerical experiments to demonstrate and validate the proposed method for sparse-view image reconstruction. Scanning and reconstruction parameters are listed in Table 1. Detector elements are equidistantly spaced at 0.127 mm.

The proposed method is compared with ASD-POCS algorithm [6] and the ADTVM algorithm [13], using the same parameters to validate their performance. Dual core is used in implementing the proposed distributed algorithm on two nodes.

In the experimental configuration, we use one detector for data acquisition by taking 36 angular samples evenly distributed over an angular range of 360°. The size of the phantom simulation is set as follows. Image size is 256 × 256 = 65536 voxels, and projection data size is 36 views, with 512 detectors or 18432 measured rays.

**Figure 1:**
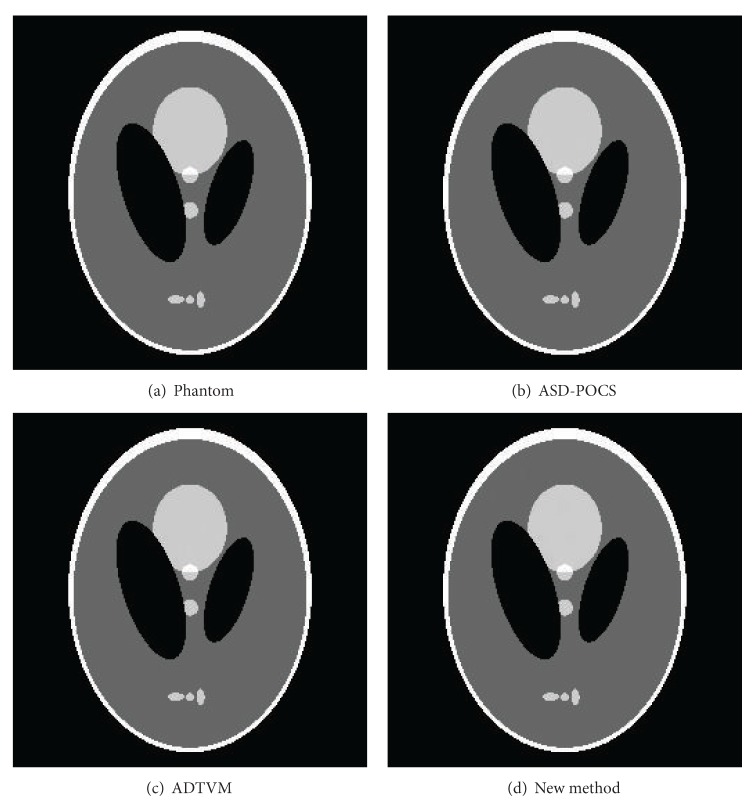
Image reconstruction of the Shepp-Logan phantom in a 36-view scan. Display window [0.1,0.35]. (a) Original image; (b) result of the ASD-POCS algorithm; (c) result of the ADTVM algorithm; (d) result of the proposed distributed algorithm.

**Figure 2:**
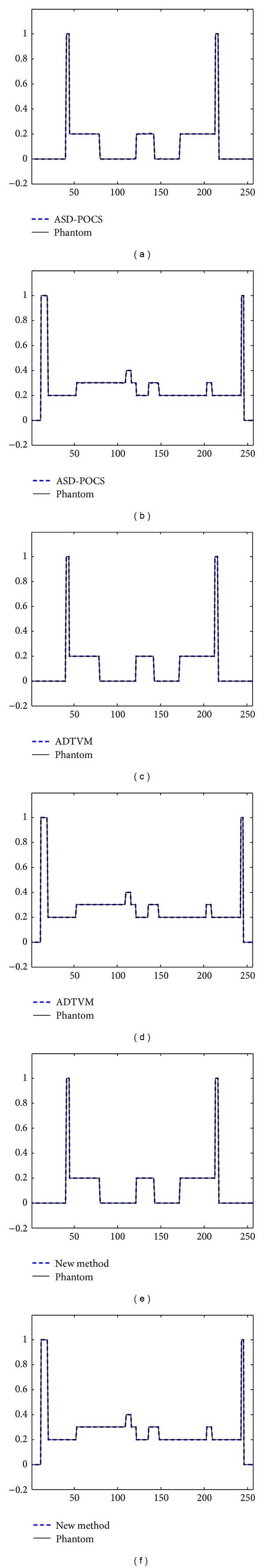
Image profiles of Figure 1. (a) Horizontal profiles along the centers of the ASD-POCS result; (b) vertical profiles along the centers of the ASD-POCS result; (c) horizontal profiles along the centers of the ADTVM result; (d) vertical profiles along the centers of the ADTVM result; (e) horizontal profiles along the centers of the proposed distributed algorithm result; (f) vertical profiles along the centers of the proposed distributed algorithm result.

The images reconstructed from this set of data using the ASD-POCS algorithm, ADTVM algorithm, and the proposed distributed algorithm are shown in Figure 1. The profiles of these images along the central horizontal and vertical rows are presented in Figure 2. The number of iterations for the three algorithms is 1000 each. The parameters of ASD-POCS are same as those in [6]. The parameters in the ADTVM algorithm and the proposed distributed algorithm are both *λ* = 1/4000 and *ρ* = 32/4000.

**Figure 3:**
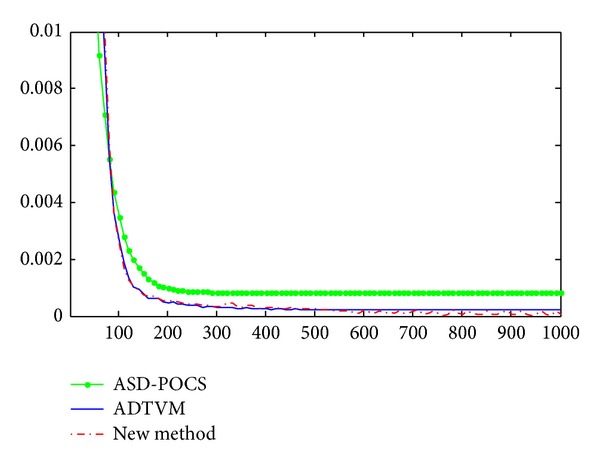
The RMSEs as functions of iterations of three tested methods.

We use the root mean squared error (RMSE) as a measure of the reconstruction error to demonstrate reconstruction accuracy quantitatively. The RMSE is defined as

(8) $$\begin{array}{c} \text{RMSE}=\sqrt{\frac{\sum_{i}\sum_{j}{\left|f\left(i,j\right)-g\left(i,j\right)\right|}^{2}}{N}}, \end{array}$$

where *f* and *g* are the ideal phantom and the reconstruction, respectively, and *N* is the total number of pixels in the image. The RMSEs of the reconstructions of the Shepp-Logan phantom are calculated. The results of the three methods are illustrated in Figure 3.  Table 2 shows the RMSE of the reconstructions from the projection data above with the three algorithms. It is clear that the accuracy and effectivity of the ADTVM algorithm and the proposed distributed algorithm are both better than those of the ASD-POCS algorithm. This is due to the use of ADM and NADA algorithms. Moreover, we can see that the accuracies of the ADTVM algorithm and proposed distributed algorithm are both almost the same. This is because the derivation and implementation of distributed algorithm are very similar as the ADTVM algorithm.

**Figure 4:**
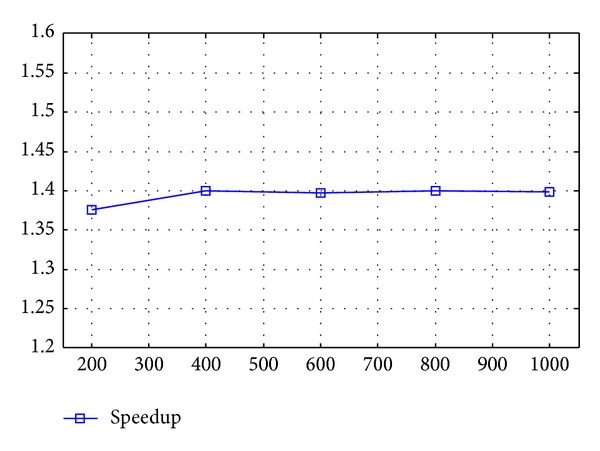
Speedup of the proposed distributed algorithm.

The running time of the three algorithms is shown in Table 3 and Figure 4 for the phantom results in the aforementioned configuration. Timing is implemented based on the average of 10 computations. The speedup is approximately 1.4 on the average.

### 3.2. Reconstruction Using Real Data

We perform experiments to reconstruct a head model from real data to further validate the proposed algorithm. Scanning and reconstruction parameters are listed in Table 4. Detector elements are equidistantly spaced at 0.635 mm.

**Figure 5:**
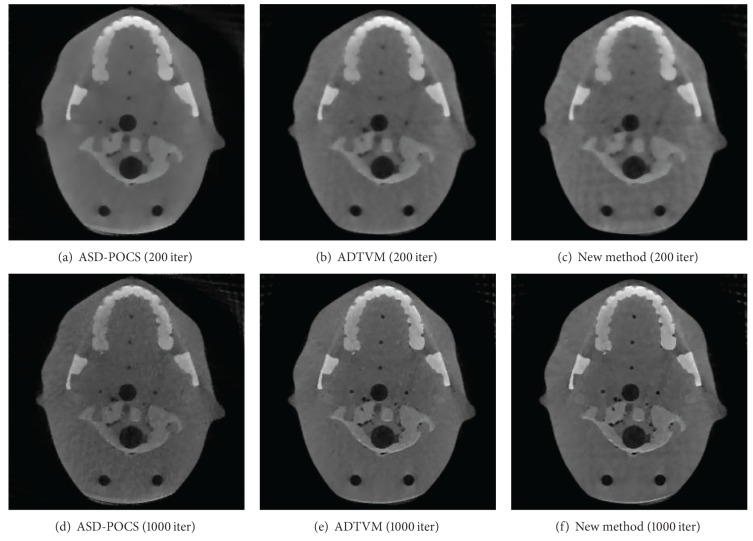
Reconstructions of the three algorithms. (a) The ASD-POCS result with 200 iterations; (b) the ADTVM result with 200 iterations; (c) the proposed distributed algorithm result with 200 iterations; (d) the ASD-POCS result with 1000 iterations; (e) the ADTVM result with 1000 iterations; (f) the proposed distributed algorithm result with 1000 iterations.

We reconstruct a *z*-axial slice for convenience. Images reconstructed using the ASD-POCS algorithm, the ADTVM algorithm, and the proposed distributed algorithm are shown in Figure 5. The numbers of iterations for the three algorithms are 200 and 1000, respectively. The experimental result suggests that ADTVM algorithm and the proposed distributed algorithm produce better reconstruction than ASD-POCS. Hence, the results of ADTVM algorithm and the proposed distributed algorithm are almost the same.

**Figure 6:**
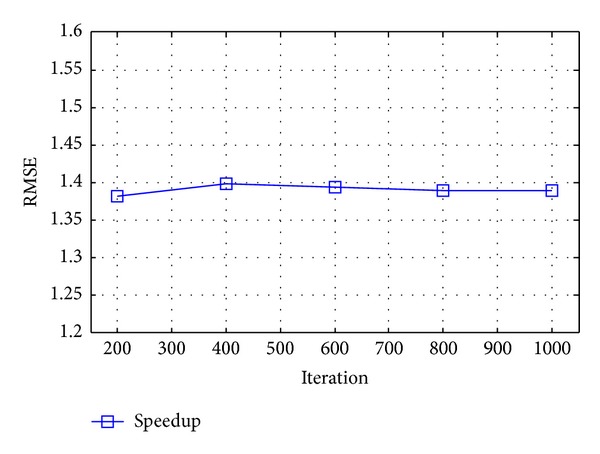
Speedup of the proposed distributed algorithm in real data reconstruction.

The running time of the three algorithms is shown in Table 5 and Figure 6 for the reconstruction results in the aforementioned configuration. Timing is implemented based on the average of 10 computations. The speedup is approximately 1.4 on the average.

We use Amdahl's law [17] to predict the theoretical maximum speedup as follows:

(9) $$\begin{array}{c} S=\frac{1}{\left(1-\beta \right)/n+\beta }, \end{array}$$

where *β* denotes the fraction of the algorithm which is strictly serial. In the ADTVM algorithm, *β* is about 0.1, so the theoretical maximum speedup in two nodes is *S* = 1.82. The speedup in real experiments will be less than *S* as the cost on communication exists in every iteration. The average speedup of the proposed distributed algorithm in all the experiments shows that the algorithm reduces the running time obviously.

All experiments are performed using C programming language under Visual Studio 2012 and OpenMP running on an AMAX Tesla workstation with Intel Xeon E5520 dual-core CPU 2.27 GHz and 24 GB memories. We partly refer to the MATLAB solver of “TVAL3” [11] for the implementation.

## 4. Conclusions

The Dis-ADTVM algorithm is as simple as the ADTVM algorithm and can accelerate the latter without accuracy loss. The new algorithm is well suited for CT image sparse-view reconstruction problem as a large-scale problem. It is clear that the Dis-ADTVM algorithm can be applied to other tomographic imaging modalities with linear system models. We will study the relationship between the performance and the number of nodes in a forthcoming paper.

## Figures and Tables

**Table 1:** Parameters in the simulation of a sparse-view scan.

Parameters	Configuration
Detector elements	512
Source to axis distance	300 mm
Source to detector distance	600 mm
Views of projection data	36
Projection data	512 × 36
Reconstruction size	256 × 256 pixels
Pixel size	0.127 × 0.127 mm^2^

**Table 2:** The RMSE of the three tested methods.

	ASD-POCS	ADTVM	Distributed algorithm
Iteration numbers	1000	1000	1000
RMSE	8.149*E* − 4	6.142*E* − 5	4.777*E* − 5

**Table 3:** Running time of the three tested methods.

Iteration number	ASD-POCS (s)	ADTVM (s)	Distributed algorithm (s)	Speedup
200	35.6462	24.1902	17.5854	1.3756
400	74.8574	51.6406	36.8742	1.4005
600	119.9293	76.7743	54.9518	1.3971
800	145.3673	103.9184	74.2152	1.4002
1000	181.7170	129.4053	92.4793	1.3993

**Table 4:** Parameters in the real data of a sparse-view scan.

Parameters	Configuration
Detector elements	640
Source to axis distance	678 mm
Source to detector distance	1610 mm
Views of projection data	72
Projection data	600 × 72
Reconstruction size	300 × 300 pixels
Pixel size	0.582 × 0.582 mm^2^

**Table 5:** Running time for reconstructing real data.

Iteration number	ASD-POCS (s)	ADTVM (s)	Distributed algorithm (s)	Speedup
200	87.2564	63.1188	45.6657	1.3822
400	184.1110	135.0983	96.6299	1.3981
600	266.1320	214.6039	153.9262	1.3942
800	356.8788	265.0989	190.7736	1.3896
1000	445.9676	334.5297	240.6383	1.3902
